# P-967. Impact of simulated ID rounds for third year pharmacy students (P3s) in an infectious diseases (ID) elective

**DOI:** 10.1093/ofid/ofae631.1157

**Published:** 2025-01-29

**Authors:** Yuman Lee, Nicole Bradley

**Affiliations:** St. John's University, Queens, New York; St. John's University, Queens, New York

## Abstract

**Background:**

ID Rounds simulate real-world multidisciplinary ID rounds for P3s enrolled in the ID elective. Two faculty play the ID attending and 4^th^ year pharmacy students on their Advanced Pharmacy Practice Experiences play the roles of the ICU fellow, nurse, microbiologist, and radiologist. An ID consult for a patient with post-influenza pneumonia along with the patient’s general information and basic labs are presented. P3s are guided to navigate the patient care process, collecting and assessing information, and formulating, implementing, and evaluating therapeutic recommendations. Challenges incorporated include a weight discrepancy, altering antimicrobials, monitoring drug levels, following cultures, and discharge planning. The purpose of this study was to assess the impact of ID Rounds on achieving Doctor of Pharmacy educational outcomes and professional activities.

**Methods:**

An anonymous, electronic survey was distributed to P3s to assess the impact of ID Rounds. Survey questions were designed in accordance with the standards set by the American Association of Colleges of Pharmacy Curriculum Outcomes and Entrustable Professional Activities, using a Likert scale. Student perceptions were also collected.

**Results:**

36 P3s responded and all strongly agreed or agreed that ID Rounds helped them learn foundational knowledge of inpatient rounds and enhanced their professional skills by building and maintaining trust among their colleagues. Majority strongly agreed or agreed that ID Rounds helped them understand the role of a pharmacist in optimizing antimicrobial use (94.5%, 34/26), become better communicators (94.5%, 34/36), and support the achievement of shared goals on a team (97.2%, 35/26). Majority rated ID Rounds a 4 out of 5 in difficulty, found the activity as an engaging way of learning about inpatient rounds, and enjoyed participating.

**Conclusion:**

ID Rounds allowed pharmacy students to achieve key standards in Doctor of Pharmacy educational outcomes and professional activities. Students responded positively to ID Rounds, which actively involved them in a real-world scenario, enabling the application of critical skills such as collaborative practice, critical thinking, and effective communication. These competencies are fundamental to the practice of pharmacy.

**Disclosures:**

**All Authors**: No reported disclosures

